# Proteomics- and Metabolomics-Based Investigation of the Effects of *Astragalus membranaceus* Powder on Meat Quality and Molecular Profiles of *Longissimus dorsi* Muscle in Ao-Hu Crossbred Rams

**DOI:** 10.3390/ani16162479

**Published:** 2026-08-10

**Authors:** Xia Lang, Yidan Xu, Bin Dao, Wangsheng Zhao, Jingjing Li, Cailian Wang

**Affiliations:** 1 Institute of Animal & Pasture Science and Green Agriculture, Gansu Academy of Agricultural Science, Lanzhou 730070, China; 2 College of Life Science and Agri-Forestry, Southwest University of Science and Technology, Mianyang 621010, China

**Keywords:** *Astragalus membranaceus* powder, *longissimus dorsi* muscle, proteomics, metabolomics

## Abstract

*Astragalus membranaceus* is a traditional Chinese medicinal herb commonly used as a feed additive in livestock production; however, the molecular mechanisms by which it influences meat quality in sheep remain poorly understood. In this study, 96 Ao-Hu crossbred rams were randomly allocated to four dietary treatments receiving 0%, 2%, 4%, or 6% *Astragalus membranaceus* powder (AMP) over a 70-day feeding trial. At the end of the trial, one sheep per pen (*n* = 6 per treatment) was selected for *longissimus dorsi* muscle collection and subsequent meat quality traits, proteomic and metabolomic analyses. Phenotypically, only the 6% AMP inclusion significantly reduced cooking loss, while all the other quality traits remained unchanged. Metabolomic analysis identified 24, 33, and 86 differential metabolites in the low-, medium-, and high-dose groups, respectively, with the high-dose group showing additional enrichment in arginine-proline metabolism, pantothenate/CoA biosynthesis, and cAMP/cGMP signaling pathways. Proteomic analysis revealed 11, 7, and 19 differentially expressed proteins, with collagen family members (*COL1A1*, *COL1A2*, and *COL3A1*) down-regulated exclusively at the high dose, though shear force was unaffected. Correlation analysis suggested associations between cooking loss and metabolites involved in fatty acid oxidation and redox balance. These findings provide clues for understanding the effects of AMP on meat quality and offer candidate pathways and protein targets for future mechanistic investigations.

## Simple Summary

Consumers today are increasingly interested in how meat is produced and what animals are fed. In this study, we tested whether adding powdered *Astragalus membranaceus*, a traditional Chinese herb, to the diet of Ao-Hu crossbred rams could improve meat quality. We divided 96 young sheep into four groups and fed them different amounts of the herb powder for 70 days, then measured meat traits and examined the molecular changes in their muscle using advanced laboratory methods. At slaughter, one sheep per pen was selected, yielding 24 animals (6 per treatment) for muscle sampling and subsequent molecular analyses. Our main finding was that only the highest dose reduced cooking loss, meaning the meat retained more moisture and remained juicier after heating. Other traits like color and tenderness did not change. At the molecular level, the high dose altered many small molecules and some proteins involved in energy and fat metabolism. Although the exact connections between these molecular shifts and cooking performance need further study, our results provide a scientific foundation for using this herb in sheep farming. This knowledge could help farmers produce lamb that cooks up more appealing, offering benefits to both producers and consumers seeking high-quality meat.

## 1. Introduction

In recent years, global demand for high-quality mutton has increased considerably, driven by growing consumer recognition of the nutritional and health benefits associated with red meat consumption [1]. Consequently, improving meat quality traits—such as tenderness, flavor, and nutritional value—has become a central objective in sheep breeding and nutrition research [2]. The Ao-Hu crossbred ram (Australian White × Hu) occupies a pivotal position in China’s mutton industry owing to its rapid growth rate, superior carcass performance, and strong adaptability [3]. However, under modern intensive farming systems, enhancing meat quality and animal health while reducing reliance on antibiotics remains a shared challenge [4].

Plant-derived feed additives have emerged as a research focus in animal nutrition due to their natural origin, low residue potential, and multifunctional properties [5]. *Astragalus membranaceus*, a traditional Chinese herbal medicine, is rich in bioactive compounds including astragalus polysaccharides, astragalosides, and flavonoids, and has been shown to exert diverse physiological functions such as immunomodulation, antioxidant activity, improved gut health, and metabolic regulation [6,7]. Recent investigations in sheep and goats have highlighted the positive impact of Astragalus on final meat quality attributes. Astragalus and its extracts have been reported to significantly improve meat color stability by increasing the relative content of oxymyoglobin and reducing metmyoglobin formation, thereby maintaining a desirable cherry-red appearance [8]. Furthermore, Astragalus feeding enhances water-holding capacity (WHC) and reduces cooking loss, likely attributed to its role in mitigating post-mortem oxidative stress and preserving muscle cell membrane integrity [8,9]. Moreover, Astragalus alters the intramuscular fatty acid profile by increasing the proportion of polyunsaturated fatty acids (PUFAs), particularly n-3 series, and enriching flavor precursors such as free amino acids and nucleotides, which collectively contribute to a more favorable sensory profile and nutritional value [3,10]. Nevertheless, while these phenotypic improvements are well-documented, the underlying molecular regulatory networks governing these changes remain largely elusive. Specifically, it remains unclear through which mechanisms—such as antioxidant defense, energy metabolism, and structural protein modulation—AMP improves meat quality, and no systematic study has integrated proteomics and metabolomics to address this in ovine muscle.

Muscle quality formation is a complex biological process orchestrated by the interplay of genetics, nutrition, and environmental factors, ultimately manifested through dynamic gene expression and metabolite accumulation [11]. Proteomics enables the systematic identification and quantification of differentially expressed proteins, revealing critical pathways related to myogenesis, energy metabolism, fiber type transformation, and antioxidant defense [12]. Complementarily, metabolomics captures real-time changes in small-molecule metabolites, directly reflecting the biochemical composition and sensory attributes of meat [13]. Integrating these multi-omics approaches offers a powerful lens to comprehensively elucidate the regulatory mechanisms through which Astragalus powder modulates muscle quality [14], thereby testing whether the observed phenotypic effects are linked to muscle metabolism and protein mechanisms. To date, few studies have systematically characterized the molecular landscape of the *longissimus dorsi* muscle in premium crossbred sheep supplemented with varying concentrations of Astragalus powder using a combined proteomic and metabolomic strategy.

In this study, Ao-Hu crossbred rams were used as experimental subjects, and different concentrations (0%, 2%, 4%, and 6%) of *Astragalus membranaceus* powder were supplemented in the diet. Proteomic and metabolomic analyses, together with measurements of conventional meat quality traits, were performed on the *longissimus dorsi* muscle to clarify the regulatory effects of *Astragalus membranaceus* powder on the muscle molecular profile and to establish a scientific basis for determining optimal supplementation levels in production. The findings are expected to provide a solid theoretical foundation and a novel scientific perspective for the application of *Astragalus membranaceus* powder as a functional feed additive in premium mutton production, thereby supporting the development of green farming strategies that integrate growth promotion with meat quality improvement.

## 2. Materials and Methods

### 2.1. Animals and Sample Preparation

This study was performed at the Lvyuanxin Meat Sheep Industrial Park located in Linxia Hui Autonomous Prefecture, Gansu Province, China, at an average altitude of 2153 m (35°25′54″ N, 103°04′59″ E), from 8 May to 27 July 2024. A total of 96 healthy Australian White × Hu crossbred male lambs, 80 days of age with an average initial body weight of 24.36 ± 0.82 kg (mean ± SD), were used. The animals were housed in a well-ventilated facility and randomly allocated to four dietary treatments receiving 0% (control, C group), 2% (low dosage, L group), 4% (medium dosage, M group), or 6% (high dosage, H group) *Astragalus membranaceus* powder (AMP; inclusion levels expressed as a percentage of the total mixed ration on an air-dry basis). Each treatment consisted of six replicate pens, with four sheep per pen. The pen served as the experimental unit for feeding management, while the individual animal (one randomly selected per pen at slaughter) served as the experimental unit for meat quality and omics analyses. The AMP was sourced from Gansu Minxian Huifeng Agricultural Technology Co., Ltd. (Lanzhou, China). Proximate analysis of AMP on a dry matter basis was performed in our laboratory following AOAC standard methods (AOAC, 2005): crude protein (Method 990.03), crude fat (Method 920.39), ash (Method 942.05), calcium (Method 968.08), and phosphorus (Method 965.17). The analysis revealed that the AMP contained 15.23% crude protein, 1.55% crude fat, 4.46% ash, 0.7% calcium, and 0.15% phosphorus. The product also contained 17.14 mg/g saponins, 4.23 mg/g flavonoids, and 159.43 mg/g astragalus polysaccharides, as determined by HPLC and UV-Vis spectrophotometry according to the Chinese Pharmacopoeia (2020 edition). The feeding trial lasted 80 days, consisting of a 10-day adaptation period followed by a 70-day formal experimental phase. Diets were formulated according to the nutritional requirements outlined in the Feeding Standard of Meat-Producing Sheep of the People’s Republic of China (NY/T816-2021). The ingredient composition and nutrient levels of the experimental diets are provided in Table S1. Before the experiment began, the pens were thoroughly cleaned and disinfected, and each lamb was numbered, ear-tagged, and dewormed. During the experimental period, all the lambs were housed indoors under a pen-fed system. Pens were cleaned and disinfected weekly. All the lambs were fed their respective experimental diets at 08:00 and 17:00 daily, with ad libitum access to fresh water throughout the experimental period. Throughout the experiment, feed intake, rumination, and general health status were closely monitored. The amount of feed offered was adjusted daily based on feed refusals, and feed intake was recorded on a pen basis. At the end of the trial, lambs were slaughtered at 160 days of age. One sheep per pen with body weight close to the pen average was randomly selected and humanely slaughtered, and the *longissimus dorsi* muscle was collected. The average pre-slaughter body weight of the four treatment groups was 38.70 ± 1.75 kg (mean ± SD).

Meat color, shear force, pH value, drip loss, press loss, and cooking loss changes were measured immediately. The remaining tissue samples were immediately snap-frozen in liquid nitrogen in cryopreservation tubes for subsequent proteomic and metabolomic analyses, then transported to the laboratory and stored at −80 °C until further processing.

### 2.2. Measurement of Meat Quality Traits

#### 2.2.1. Meat Color

*Longissimus dorsi* muscle samples were taken, the surface fascia removed, and the samples stored in a 4 °C refrigerator after 45 min and 24 h of storage. Prior to measurement, the CR-410 color difference meter (Konica Minolta, Tokyo, Japan) was calibrated. The lightness (L*), redness (a*) and yellowness (b*) values of the muscle were measured. For each sample, two muscle pieces were used, and three readings were taken at three different points on each piece; the average value was calculated.

#### 2.2.2. Shear Force

After storage at 4 °C for 24 h, muscle samples were trimmed of visible fascia and fat, and specimens of 3 cm × 1 cm × 1 cm were cut parallel to the muscle fibre direction. Samples were heated in a water bath at 85 °C until the core temperature reached 70 °C, then cooled to room temperature. Shear force was measured using a muscle tenderness tester (RH-N50, Guangzhou Runhu, Guangzhou, China), with the results expressed in Newtons (N). Each sample was measured three times and the average value was recorded.

#### 2.2.3. pH

The pH values of muscle samples were measured at 45 min and 24 h postmortem using a calibrated portable pH meter (testo-205, Testo AG, Titisee-Neustadt, Germany). The electrode was inserted into the sample at three different points, and the average value was recorded at each time point. The pH meter was calibrated with standard buffer solutions before each use.

#### 2.2.4. Drip Loss

The fascia and visible fat on the sample surface were removed, and the muscle was trimmed into a strip of approximately 5 cm × 3 cm × 2 cm, the initial weight of which was recorded as W_1_. The trimmed sample was then suspended at the center of a self-sealing bag with fine wire, with care taken to avoid contact between the sample and the bag walls. After storage at 4 °C for 24 h, the sample was reweighed (W_2_). Drip loss was calculated using the following formula:

Drip loss (%) = (W_1_ − W_2_)/W_1_ × 100, (1)

#### 2.2.5. Press Loss

The collected samples were stored at 4 °C for 24 h. Subsequently, 1 cm thick muscle slices were cut using a circular cutter (3 cm diameter). The initial weight (W_3_) was accurately recorded using an analytical balance. Each muscle slice was placed between 18 layers of neutral qualitative filter paper on each side and subjected to a pressure of 35 kg for 5 min using a meat press. After removing the pressure, the sample was reweighed (W_4_). Press loss (%) was calculated as

Press loss (%) = (W_3_ − W_4_)/W_3_ × 100, (2)

#### 2.2.6. Cooking Loss

Approximately 50 g of muscle sample (W_5_) was placed in a self-sealing bag, sealed, and heated in a water bath at 75 °C for 45 min. After heating, the sample was removed, cooled at room temperature for 15 min, and reweighed (W_6_). Cooking loss (%) was calculated as

Cooking loss (%) = (W_5_ − W_6_)/W_5_ × 100, (3)

### 2.3. Metabolomic Analysis

#### 2.3.1. Sample Preparation

For metabolomic analysis, an appropriate amount of each sample was accurately weighed into a 2 mL centrifuge tube, and 1000 µL of tissue extract (75% methanol:chloroform, 9:1, *v*/*v*, mixed with 25% water) was added together with steel balls. The samples were homogenized using a tissue grinder at 50 Hz for 60 s, and the grinding step was repeated twice. The homogenates were then subjected to ultrasonication at room temperature for 30 min, followed by ice-bath incubation for 30 min. After centrifugation at 12,000 rpm for 10 min at 4 °C, the supernatant was transferred to a new 2 mL centrifuge tube and concentrated to dryness under vacuum. The dried residues were reconstituted in 200 µL of 50% acetonitrile containing 2-chloro-L-phenylalanine (4 ppm) as an internal standard. The resulting solutions were filtered through a 0.22 μm membrane and transferred into autosampler vials for LC-MS analysis.

#### 2.3.2. Liquid Chromatograph Tandem Mass Spectrometer (LC-MS/MS) Analysis

Chromatographic separation was performed on a Vanquish UHPLC System (Thermo Fisher Scientific, Waltham, MA, USA) equipped with an ACQUITY UPLC^®^ HSS T3 column (2.1 × 100 mm, 1.8 μm; Waters, Milford, MA, USA) maintained at 40 °C. The flow rate was set at 0.3 mL/min, and the injection volume was 2 μL. For positive ion mode analysis, the mobile phases consisted of (A2) 0.1% formic acid in water and (B2) 0.1% formic acid in acetonitrile, with the following gradient elution: 0–1 min, 10% B2; 1–5 min, 10–98% B2; 5–6.5 min, 98% B2; 6.5–6.6 min, 98–10% B2; 6.6–8 min, 10% B2. For negative ion mode analysis, the mobile phases consisted of (A3) 5 mM ammonium formate and (B3) acetonitrile, with the gradient as follows: 0–1 min, 10% B3; 1–5 min, 10–98% B3; 5–6.5 min, 98% B3; 6.5–6.6 min, 98–10% B3; 6.6–8 min, 10% B3.

Mass spectrometric detection was performed on an Orbitrap Exploris 120 mass spectrometer (Thermo Fisher Scientific, Waltham, MA, USA) equipped with an electrospray ionization (ESI) source operating in both positive and negative ion modes. Data were acquired using a full MS/data-dependent MS/MS (ddMS2) mode. The key parameters were set as follows: sheath gas pressure, 40 arb; auxiliary gas flow, 10 arb; spray voltage, 3.50 kV (positive) and −2.50 kV (negative); capillary temperature, 325 °C; MS1 scan range, *m*/*z* 100–1000; MS1 resolution, 60,000 FWHM; number of data-dependent scans per cycle, 4; MS/MS resolution, 15,000 FWHM; normalized collision energy, 30%; dynamic exclusion time, automatic.

#### 2.3.3. Bioinformatic Analysis

Raw data were converted to mzXML format using Proteowizard (v3.0.8789). Peak detection, filtering, and alignment were performed using XCMS (v3.12.0) with parameters: bw = 2, ppm = 15, peakwidth = c (5, 30), mzwid = 0.015, mzdiff = 0.01, method = “centWave”. Metabolite identification was conducted by matching accurate mass and MS/MS spectra against the following databases: HMDB, MassBank, KEGG, LipidMaps, mzCloud, and an in-house library (Panomix Biomedical Tech Co., Ltd., Suzhou, China). All the metabolite annotations were based on MS/MS spectral matching against public databases and should therefore be considered putative identifications. Multivariate data analysis was performed using the ropls package in R. Unit variance scaling was applied to the data prior to modeling. Principal component analysis (PCA) was first conducted to visualize overall clustering trends and detect potential outliers. Partial least squares-discriminant analysis (PLS-DA) and orthogonal partial least squares-discriminant analysis (OPLS-DA) were subsequently performed to maximize the separation between groups. The model’s robustness was evaluated by a permutation test (*n* = 200), and the goodness-of-fit was reported as R^2^Y (cum), while the predictive ability was reported as Q^2^ (cum). Metabolites with |log_2_ fold change| > 1, VIP > 1, and *p*-value < 0.05 were considered statistically significant differential metabolites. Given the exploratory nature of this study, no formal multiple-testing correction was applied; instead, the combined criteria of fold change, VIP, and *p*-value were adopted to prioritize metabolites with both statistical significance and biological relevance, while acknowledging that the identified candidates require further validation. Kyoto Encyclopedia of Genes and Genomes (KEGG) pathway enrichment analysis was performed using a hypergeometric distribution-based enrichment method [15], and KEGG Mapper was used for visualization.

### 2.4. Proteome Analysis

#### 2.4.1. Protein Extraction and Digestion

Protein extraction was performed using the SDT lysis buffer (4% SDS, 100 mM Tris-HCl, pH 7.6). A total of 24 muscle samples were processed for protein extraction. Briefly, samples were homogenized using an MP FastPrep-24 homogenizer (24 × 2 cycles, 6.0 m/s, 60 s per cycle). Following homogenization, SDT buffer was added, and the lysates were boiled for 15 min. After centrifugation at 14,000× *g* for 40 min, the supernatant was collected, and protein concentration was determined using a BCA Protein Assay Kit (Bio-Rad, Hercules, CA, USA). For quality control, 15 µg of protein from each sample was mixed with 5× loading buffer, boiled for 5 min, and separated on 4–20% SDS-PAGE gels at a constant voltage of 180 V for 45 min. Protein bands were visualized by Coomassie Blue R-250 staining. For protein digestion, dithiothreitol (DTT) was added to each sample at a final concentration of 40 mM and incubated at 600 rpm for 1.5 h at 37 °C to reduce disulfide bonds. After cooling to room temperature, iodoacetamide (IAA) was added to a final concentration of 20 mM and the mixture was incubated in the dark for 30 min to alkylate cysteine residues. The samples were then transferred to 10 kDa Microcon filters (Millipore, Burlington, MA, USA). The filters were washed three times with 100 µL of UA buffer (8 M urea, 100 mM Tris-HCl, pH 8.5) and twice with 100 µL of 25 mM NH_4_HCO_3_ buffer. Trypsin was added at a 1:50 (trypsin:protein, *w*/*w*) ratio, and digestion was carried out at 37 °C for 15–18 h. The resulting peptides were collected by centrifugation as filtrate.

#### 2.4.2. LC-MS/MS Analysis

Peptides were desalted using C18 Cartridges (Empore™ SPE Cartridges MCX, 30 µm, Waters), concentrated by vacuum centrifugation, and reconstituted in 40 µL of 0.1% (*v*/*v*) formic acid. Peptide concentration was estimated by measuring UV absorbance at 280 nm. For data-independent acquisition (DIA) analysis, indexed retention time (iRT) calibration peptides were spiked into each sample. LC-MS/MS analysis was performed on an Orbitrap™ Astral™ mass spectrometer (Thermo Scientific, Waltham, MA, USA) coupled to a Vanquish Neo liquid chromatography system (Thermo Scientific) operating in DIA mode. Precursor ions were scanned over a mass range of 380–980 *m*/*z* with a resolution of 240,000 at 200 *m*/*z*. A total of 299 windows were set for MS2 scanning with an isolation window of 2 *m*/*z*. Higher-energy collisional dissociation (HCD) fragmentation was applied at 25 eV. The normalized automatic gain control (AGC) target was set to 500% with a maximum injection time of 5 ms for MS1 and 3 ms for MS2. DIA data were processed using DIA-NN (version 1.8.1). The main parameters were set as follows: enzyme, trypsin; maximum missed cleavages, 1; fixed modification, carbamidomethyl (C); variable modifications, oxidation (M) and acetylation (protein N-terminus).

#### 2.4.3. Bioinformatic Analysis

DIA data were processed using DIA-NN (version 1.8.1) against the UniProt sheep (Ovis aries) protein database. Search parameters were: enzyme, trypsin; maximum missed cleavages, 1; fixed modification, carbamidomethyl (C); variable modifications, oxidation (M) and acetylation (protein N-terminus); precursor FDR ≤ 1%. The same multivariate analysis methods as described for metabolomics were used. Differentially expressed proteins (DEPs) were defined as those exhibiting an absolute |log_2_ fold change| > 1, VIP > 1, and *p*-value < 0.05. KEGG pathway annotations were performed on the target protein set using KOBAS software (version 3.0). Fisher’s exact test was employed to compare the distribution of each KEGG pathway between the target protein set and the overall protein set, and enrichment analysis was conducted on the target protein set for KEGG pathway annotations.

### 2.5. Correlation Analysis

Pair-wise Spearman’s correlation coefficients were calculated among proteomics, metabolomics, and meat quality traits using R software (version 3.5.1) [11]. Selection criteria were set as an absolute value of the correlation coefficient greater than 0.6 (|r| > 0.6) and a *p*-value < 0.05. Cytoscape_v3.10.3 was utilized to build the network diagram relationship among these. The R package complex_heat_map was used to visualize the correlation coefficients using heatmap plots.

### 2.6. Statistical Analysis

The results of this study were expressed as the mean ± standard error of the mean (SEM). Prior to ANOVA, the assumptions of normality (Shapiro–Wilk test) and homogeneity of variances (Levene’s test) were verified for all meat quality traits. All data met these assumptions (*p* > 0.05). When significant differences were detected, Tukey’s honestly significant difference (HSD) post hoc test was applied for multiple comparisons among treatment groups. For the omics data, Student’s *t*-test was used for pairwise comparisons between each AMP group and the control group. SPSS 22.0 software (SPSS, Inc., Chicago, IL, USA) was used to perform a one-way analysis of variance (ANOVA) at a 95% confidence level (α = 0.05). Data visualization was accomplished with Origin 2024 (version 2024, OriginLab, Northampton, MA, USA). Additionally, MetaboAnalyst 6.0 (Xia Lab, McGill University, Montreal, QC, Canada) was utilized to analyze the heatmaps and various correlation analyses. Statistical significance was set at *p*-value < 0.05 or *p*-value < 0.01.

## 3. Results

### 3.1. Meat Quality Traits

As shown in Table 1, the addition of 6% *Astragalus membranaceus* powder to the diet significantly reduced the cooking loss of the *longissimus dorsi* muscle in Ao-hu crossbred rams (*p* < 0.05), while no significant differences were observed in the other meat quality indicators, including meat color, shear force, pH, drip loss, and press loss (*p* > 0.05). Thus, the phenotypic response to AMP was both trait-specific and limited to the highest inclusion level tested.

### 3.2. Metabolomics Analysis

**Figure 1:**
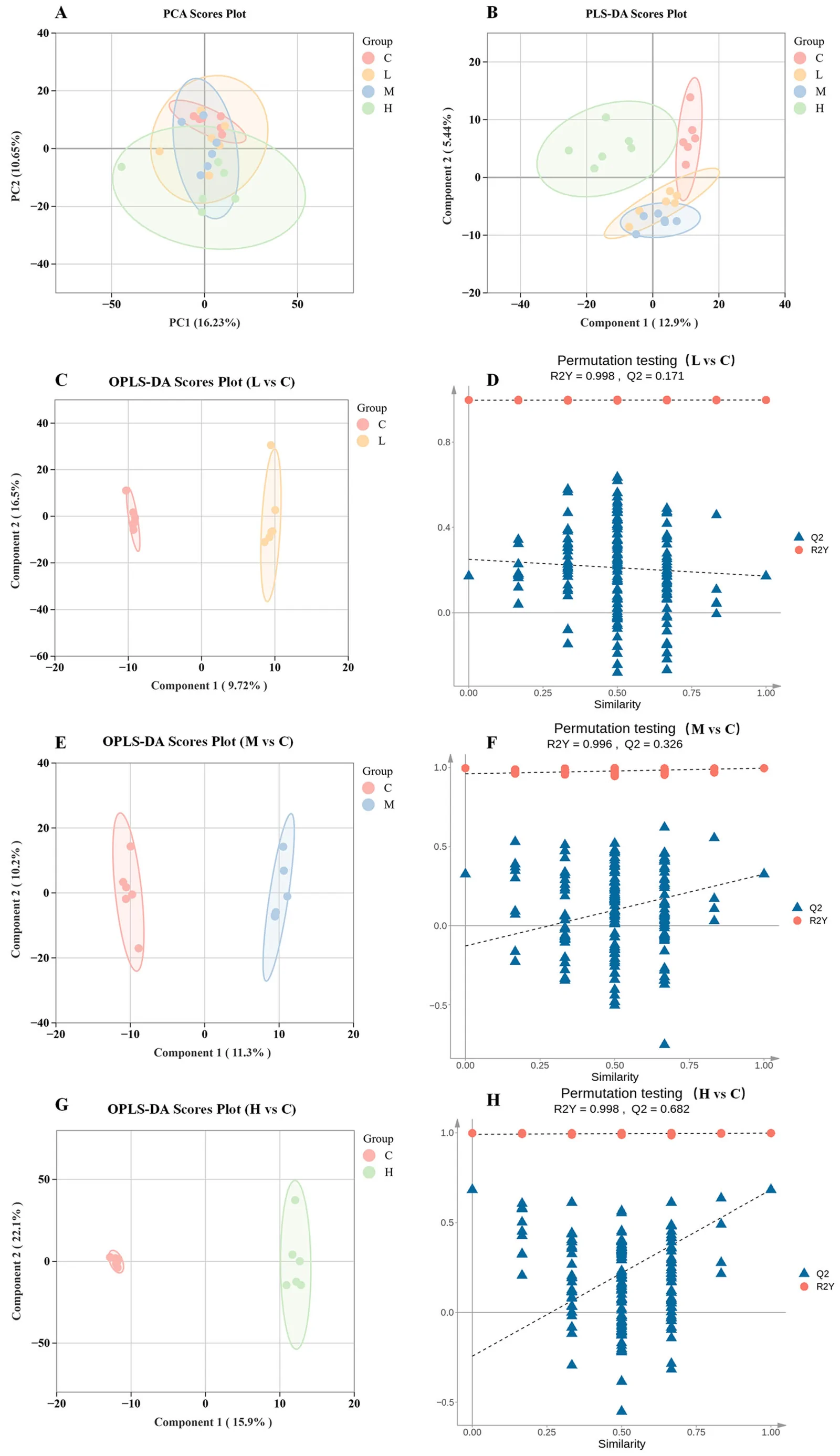
Results of multivariate statistical analysis of metabolomics data from the *longissimus dorsi* muscle: (**A**) PCA model score plots, (**B**) PLS-DA model score plots, (**C**,**E**,**G**) OPLS-DA model score plots, and (**D**,**F**,**H**) permutation test plots. The abbreviations L, M, H and C represent the low-dose group, medium-dose group, high-dose group, and control group, respectively.

**Figure 2:**
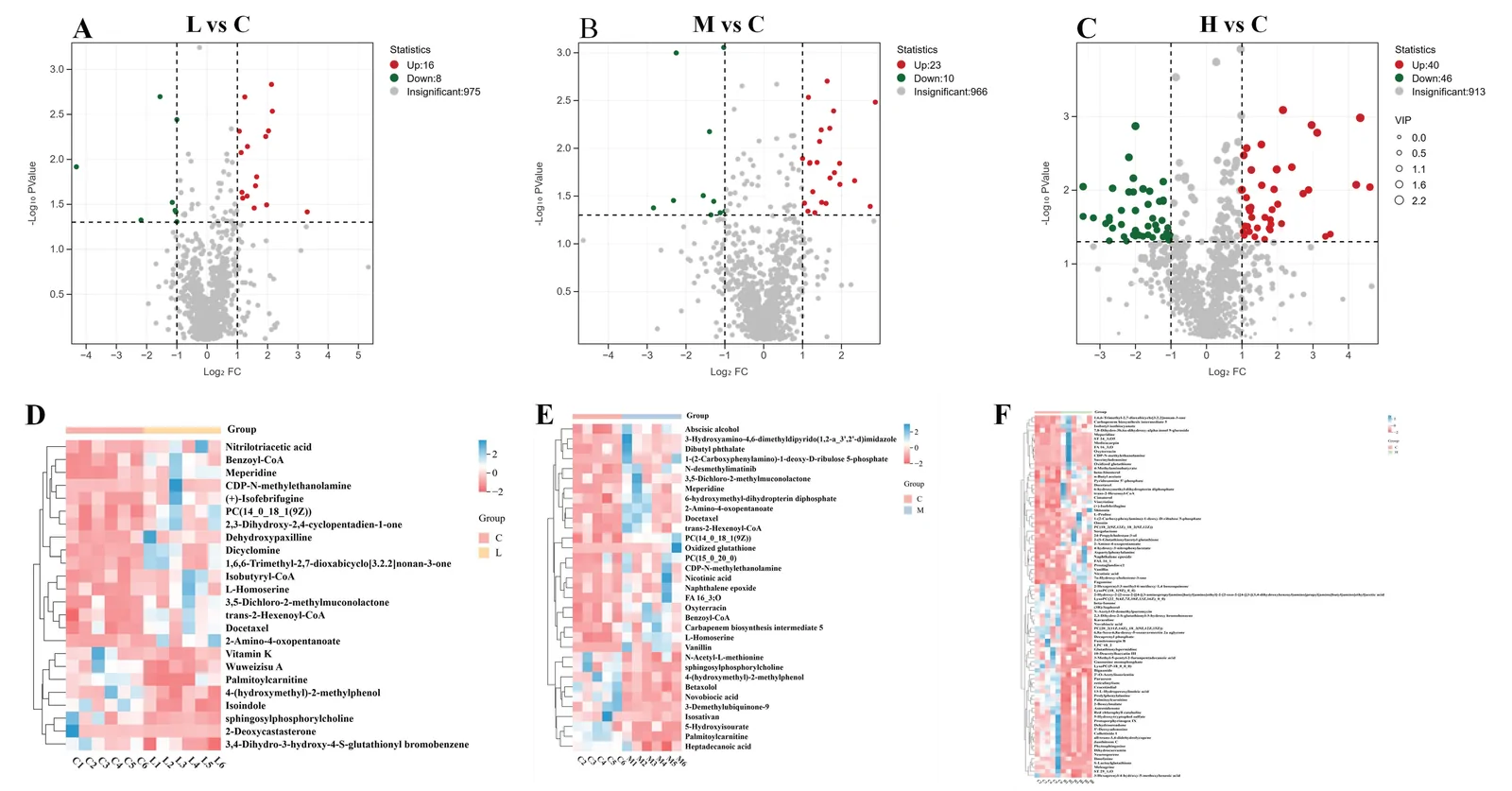
Volcano plots and heatmaps showing differential metabolites between the C group and the AMP-supplemented groups. (**A**–**C**) show the volcano plots for the L group, M group, and H group, respectively, compared to the C group; (**D**–**F**) show the heatmaps for the L, M, and H groups, respectively, compared to the C group. The abbreviations L, M, H and C represent the low-dose group, medium-dose group, high-dose group, and control group, respectively.

Under the combined anion-cation mode, the identified metabolite data were analyzed using PCA and PLS-DA methods, and the results are presented in Figure 1A,B, respectively. The OPLS-DA model showed clear separation between the AMP-supplemented groups (L, M, and H) and the C group, indicating that dietary AMP significantly affects the metabolism of the *longissimus dorsi* muscle in Ao-hu crossbred rams (Figure 1C,E,G). The R^2^Y and Q^2^ values for each group demonstrated that the model possesses good explanatory and predictive capabilities (Figure 1D,F,H). Among the three comparisons, the H vs. C model showed moderate predictive ability (Q^2^ = 0.682), followed by the M vs. C model (Q^2^ = 0.326), while the L vs. C model exhibited low predictive power (Q^2^ = 0.171). A total of 24 differential metabolites (16 up-regulated and 8 down-regulated) were identified in the L group (Figure 2A); 33 differential metabolites (23 up-regulated and 10 down-regulated) were identified in the M group (Figure 2B); the number of differential metabolites in the H group increased significantly, with a total of 86 differential metabolites identified (40 up-regulated and 46 down-regulated) (Figure 2C). Figure 2D–F show the differential metabolites in each comparison group.

**Figure 3:**
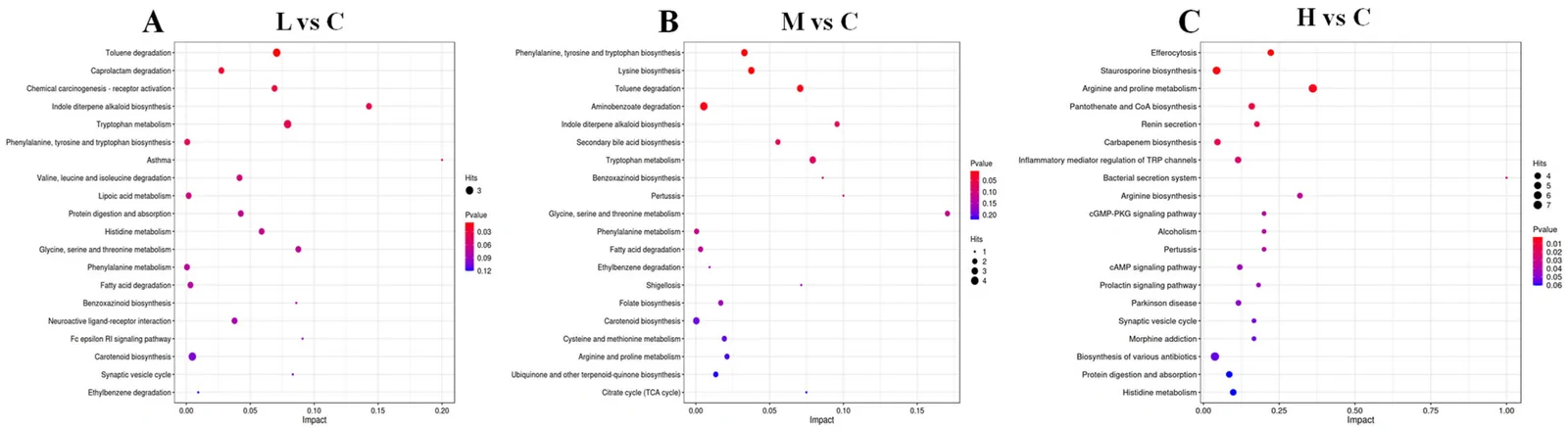
KEGG pathway enrichment bubble plots of differential metabolites between the C group and the AMP-supplemented groups. (**A**–**C**) represent the top metabolic pathways enriched in the L vs. C, M vs. C, and H vs. C groups, respectively. The abbreviations L, M, H and C represent the low-dose group, medium-dose group, high-dose group, and control group, respectively.

The KEGG pathway enrichment analysis results indicated that the key pathways regulated by different levels of *Astragalus membranaceus* powder differed when compared with the C group. Specifically, compared with C group, L group was primarily enriched in 7 pathways: toluene degradation, caprolactam degradation, chemical carcinogenesis—receptor activation, indole diterpene alkaloid biosynthesis, tryptophan metabolism, phenylalanine, tyrosine and tryptophan biosynthesis, and asthma (*p* < 0.05) (Figure 3A); M group was primarily enriched in 4 pathways: phenylalanine, tyrosine and tryptophan biosynthesis, lysine biosynthesis, toluene degradation, and aminobenzoate degradation (*p* < 0.05) (Figure 3B); H group was primarily enriched in 14 pathways: efferocytosis, staurosporine biosynthesis, arginine and proline metabolism, pantothenate and CoA biosynthesis, renin secretion, carbapenem biosynthesis, inflammatory mediator regulation of TRP channels, bacterial secretion system, arginine biosynthesis, cGMP-PKG signaling pathway, alcoholism, pertussis, cAMP signaling pathway, Prolactin signaling pathway, and parkinson disease (*p* < 0.05) (Figure 3C)

### 3.3. Proteomics Analysis

**Figure 4:**
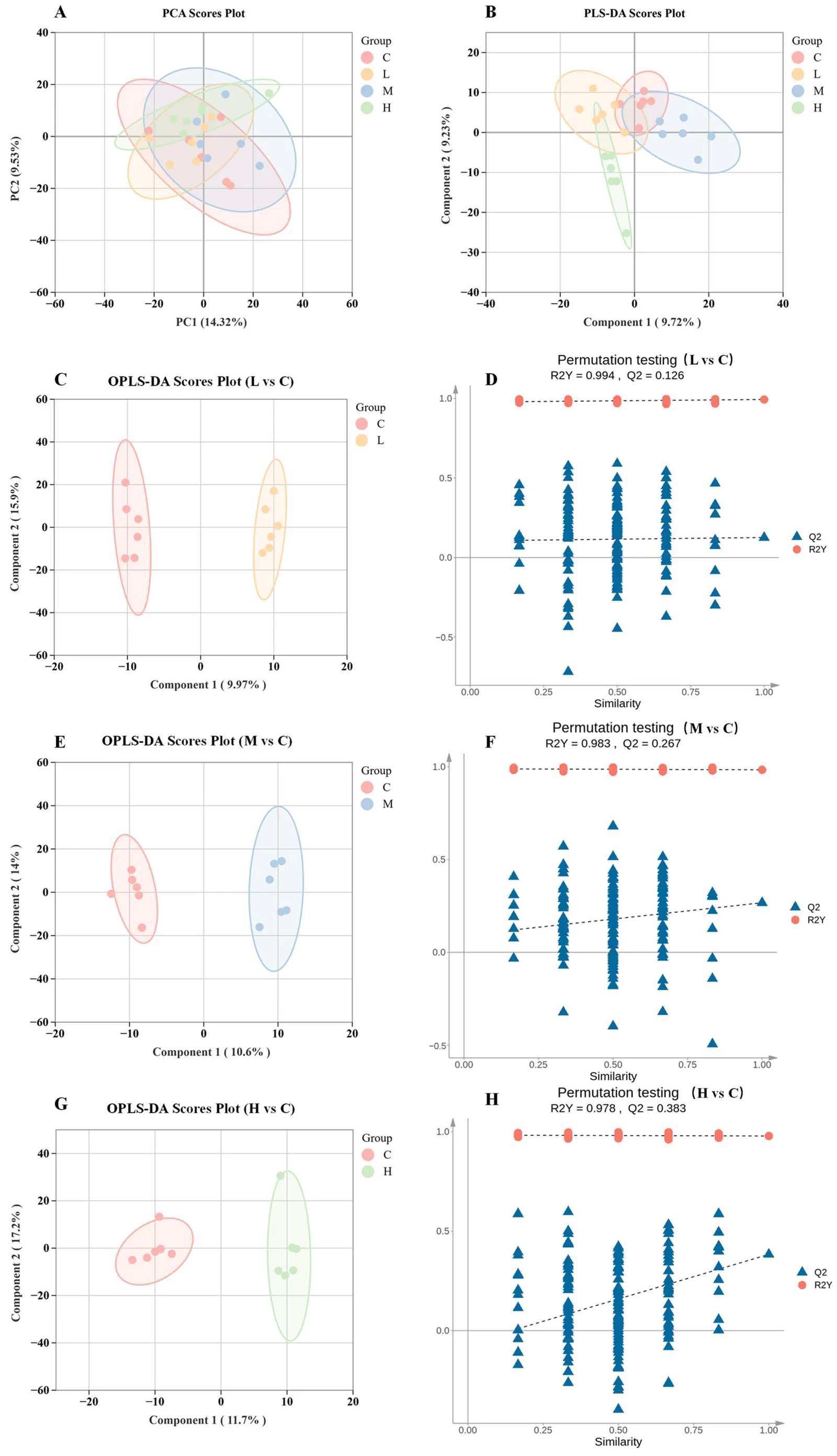
Results of multivariate statistical analysis of proteomics data from the *longissimus dorsi* muscle: (**A**) PCA model score plots, (**B**) PLS-DA model score plots, (**C**,**E**,**G**) OPLS-DA model score plots, and (**D**,**F**,**H**) permutation test plots. The abbreviations L, M, H and C represent the low-dose group, medium-dose group, high-dose group, and control group, respectively.

**Figure 5:**
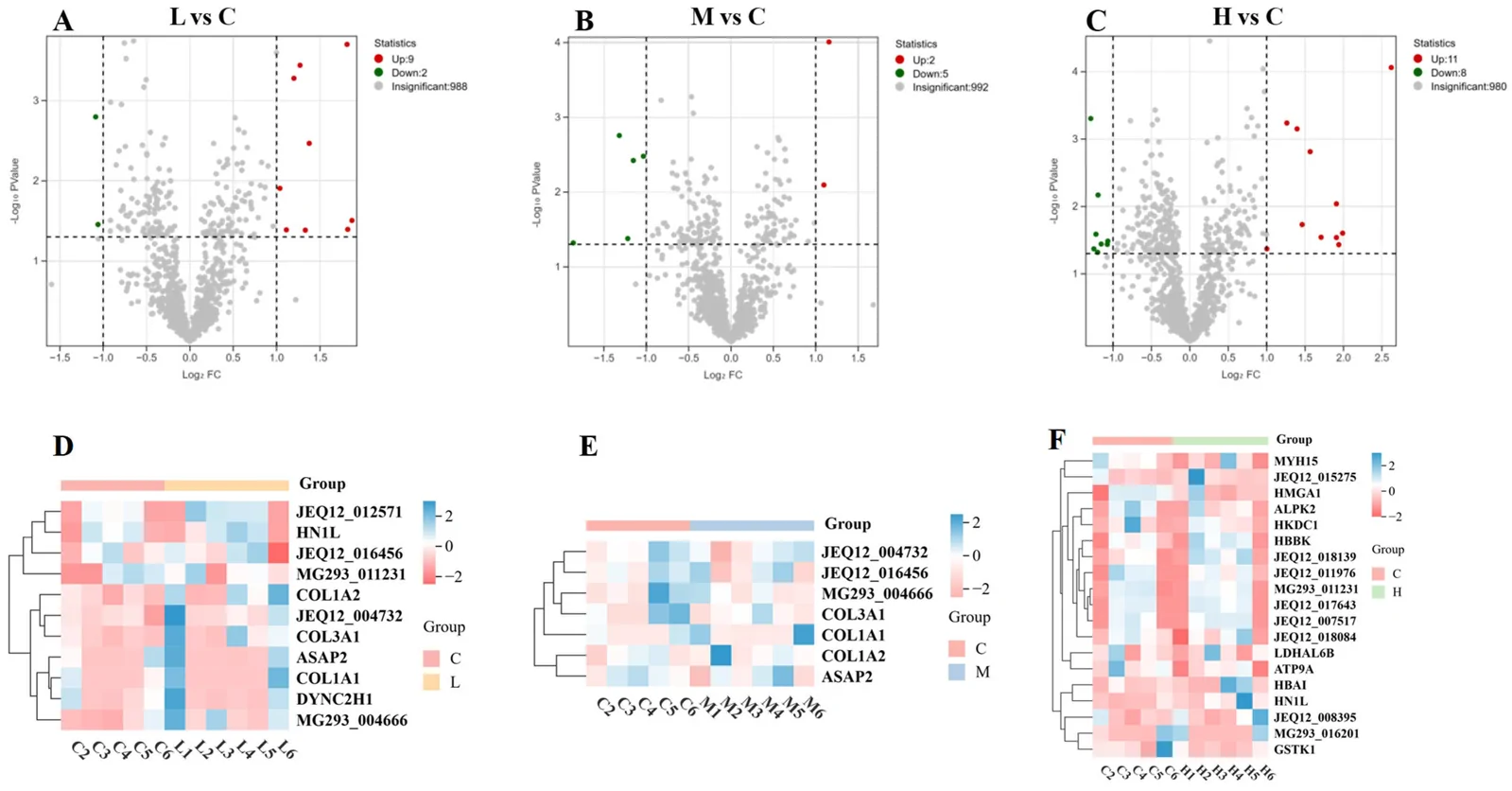
Volcano plots and heat-maps showing DEPs between the C group and the AMP-supplemented groups. (**A**–**C**) show volcano plots for the L group, M group, and H group, respectively, compared to the C group; (**D**–**F**) show heatmaps for the L, M, and H groups, respectively, compared to the C group. The abbreviations L, M, H and C represent the low-dose group, medium-dose group, high-dose group, and control group, respectively.

**Figure 6:**
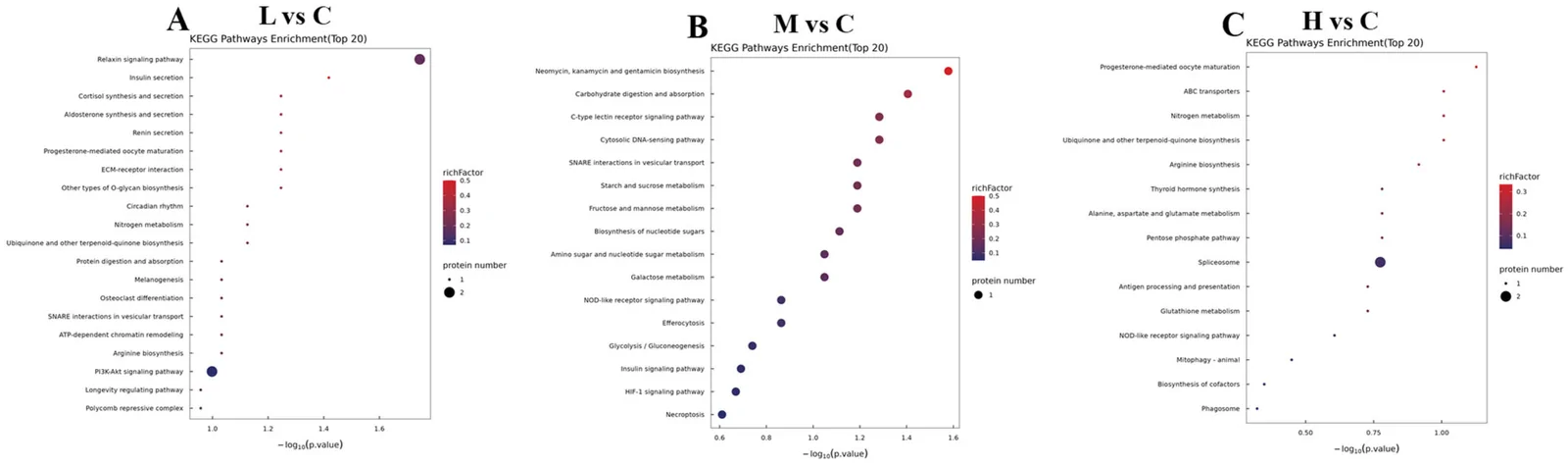
KEGG pathway enrichment bubble plots of DEPs between the C group and the AMP-supplemented groups. (**A**–**C**) represent the top metabolic pathways enriched in the L vs. C, M vs. C, and H vs. C groups, respectively. The abbreviations L, M, H and C represent the low-dose group, medium-dose group, high-dose group, and control group, respectively.

The identified protein data were analyzed using PCA and PLS-DA methods, and the results are presented in Figure 4A,B. PCA showed substantial overlap among groups, indicating that global proteomic differences were relatively modest. PLS-DA revealed clear separation of the H groups from the C group, whereas the M and L groups showed only partial separation, suggesting that the proteomic response to AMP supplementation was more pronounced at higher inclusion levels. To further validate the effects of *Astragalus membranaceus* powder supplementation on the proteomic profiles of the *longissimus dorsi* muscle in Ao-hu crossbred rams, a supervised OPLS-DA model was subsequently constructed. As shown in Figure 4C,E,G, the three comparison groups exhibit distinct and well-resolved clustering in the principal component projection space, with no overlap. The R^2^Y and Q^2^ values of the OPLS-DA permutation tests are presented in Figure 4D,F,H. Among the three comparisons, the H vs. C model showed moderate predictive ability (Q^2^ = 0.383), followed by the M vs. C model (Q^2^ = 0.267), while the L vs. C model exhibited low predictive power (Q^2^ = 0.126). Based on the screening criteria of |log_2_ fold change| > 1, VIP > 1, and *p*-value < 0.05, a total of 11 DEPs, including *COL1A2*, *COL1A1*, *JEQ12_004732*, *JEQ12_016456*, *COL3A1*, *ASAP2*, *MG293_004666*, *JEQ12_012571*, *DYNC2H1*, *MG293_011231*, and *HN1L*, were identified in the C vs. L comparison. (Figure 5A). In the C vs. M comparison, 7 DEPs (*COL1A2*, *COL1A1*, *JEQ12_004732*, *JEQ12_016456*, *COL3A1*, *ASAP2*, and *MG293_004666*) were identified based on the same criteria (Figure 5B). In the C vs. H comparison, the number of DEPs increased significantly, with a total of 19 DEPs identified (11 up-regulated and 8 down-regulated) (Figure 5C). The DEPs in each group were visualized using heatmaps (Figure 5D–F). KEGG pathway enrichment analysis was performed for each comparison group. The C vs. L group showed significant enrichment in pathways related to relaxin signaling pathway and insulin secretion (*p* < 0.05) (Figure 6A); the C vs. M group was dominated by neomycin, kanamycin and gentamicin biosynthesis and carbohydrate digestion and absorption (*p* < 0.05) (Figure 6B); while the C vs. H comparison, no KEGG pathway reached statistical significance (Figure 6C).

### 3.4. Correlation Analysis

**Figure 7:**
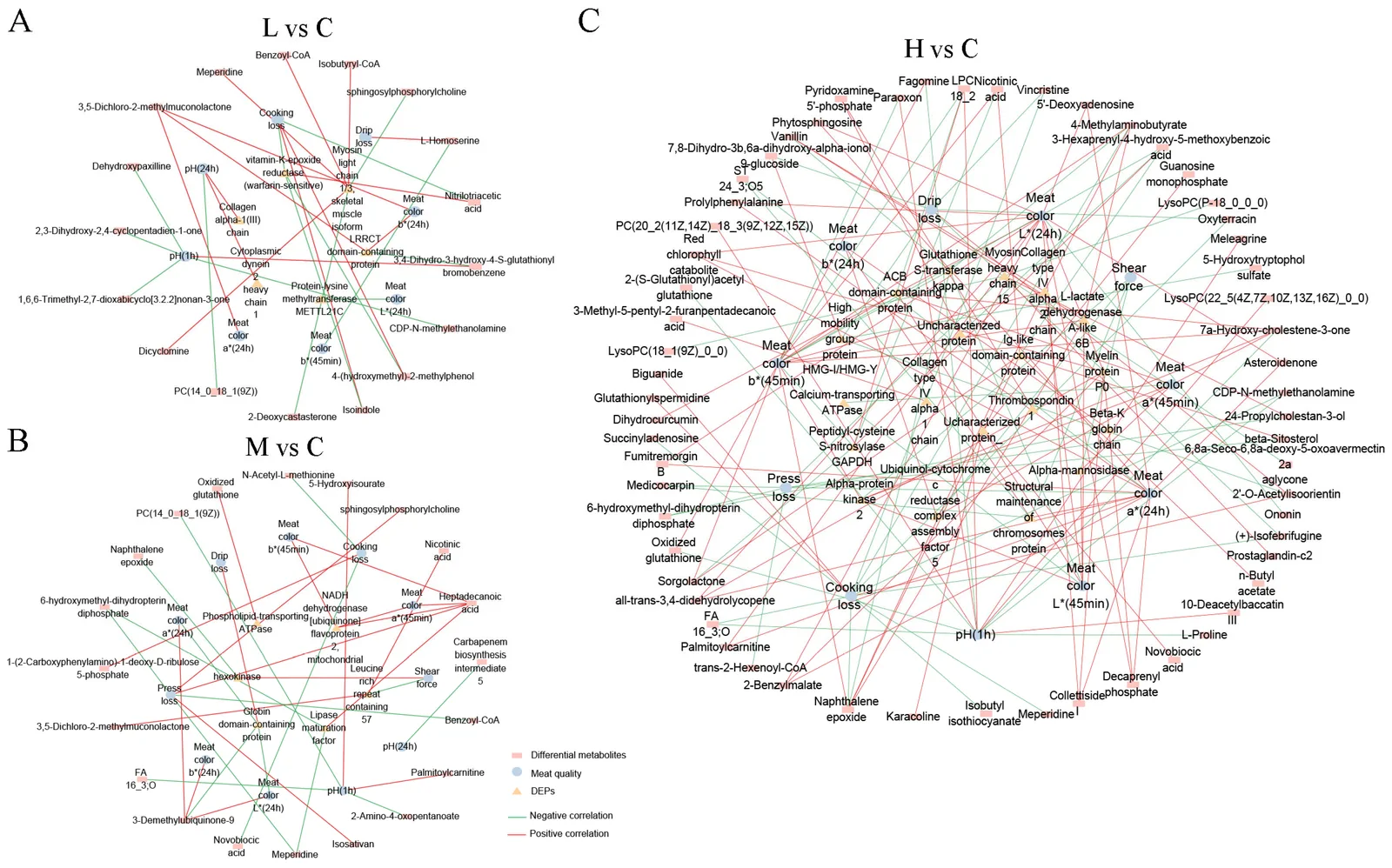
Correlation networks of differential metabolites, DEPs, and meat quality. (**A**–**C**) show the correlation networks of differentially expressed metabolites, proteins, and meat quality for the L, M, and H groups, respectively, compared to the C group. Pink squares represent differential metabolites, blue circles represent meat quality, and yellow triangles represent DEPs. Red line indicates a positive correlation, while green line represents a negative correlation between variables. The abbreviations L, M, H and C represent the low-dose group, medium-dose group, high-dose group, and control group, respectively.

In the L group, which lacked significant phenotypic changes, multiple correlations were observed among structural proteins, metabolites, and meat quality traits, including Myosin light chain 1/3, pH (1 h), cooking loss, and vitamin-K-epoxide reductase (Figure 7A). However, as these associations were not accompanied by corresponding phenotypic improvements, they are not discussed further in terms of mechanistic interpretation.

Significant correlations were also observed in the M group (Figure 7B) (*p* < 0.05). Press loss was positively correlated with isosativan, heptadecanoic acid, and sphingosylphosphorylcholine, and negatively correlated with meperidine, 6-hydroxymethyl-dihydropterin diphosphate, and benzoyl-CoA. Heptadecanoic acid was additionally correlated with lipase maturation factor, NADH dehydrogenase [ubiquinone] flavoprotein 2, mitochondrial, meat color a* (45 min), meat color b* (45 min), and press loss. pH (1 h) showed positive correlations with 5-hydroxyisourate and palmitoylcarnitine, and negative correlations with 2-amino-4-oxopentanoate, FA 16:3;O, and PC (14:0_18:1 (9Z)). 3-demethylubiquinone-9 was positively associated with meat color a* (24 h), meat color b* (24 h), and meat color L* (24 h), and negatively with globin domain-containing protein, which was positively correlated with drip loss and negatively with meat color a* (24 h) and meat color L* (24 h).

In the H group, for instance, meat color b* (45 min) was positively correlated with nicotinic acid, oxidized glutathione, l-lactate dehydrogenase a-like 6b, and collagen type IV alpha 2 chain, and negatively correlated with LPC 18_2 and glutathione s-transferase kappa. Meat color L* (24 h) was positively correlated with phytosphingosine, palmitoylcarnitine, dihydrocurcumin, all-trans-3,4-didehydrolycopene, prolylphenylalanine, and 5′-deoxyadenosine, and negatively correlated with ononin and ST 24_3;O5. Meat color a* (24 h) was positively correlated with 2-benzylmalate, 5′-deoxyadenosine, prolylphenylalanine, palmitoylcarnitine, all-trans-3,4-didehydrolycopene, and collettiside I, and negatively correlated with ST 24_3;O5 and medicocarpin. Besides, cooking loss was positively correlated with glutathionylspermidine, 5′-deoxyadenosine, prolylphenylalanine, palmitoylcarnitine, and phytosphingosine, and negatively correlated with naphthalene epoxide, isobutyl isothiocyanate, FA 16_3;O, and meperidine. Conversely, pH (1 h) showed positive correlations with LPC 18_2, paraoxon, meleagrine, 10-deacetylbaccatin III, and dehydrooreadone, and negative correlations with L-proline, oxidized glutathione, FA 16_3;O, CDP-n-methylethanolamine, and 2-(s-glutathionyl)acetyl glutathione. Notably, several other meat quality traits, such as shear force, press loss, and drip loss, were also measured in the samples; however, they did not show as many significant correlations with the key metabolites as the aforementioned traits (Figure 7C).

## 4. Discussion

Consumer demand for high-quality mutton has grown considerably in recent years, and plant-derived feed additives have attracted increasing interest as modulators of meat quality attributes. Among these, *Astragalus membranaceus*—a traditional Chinese medicinal herb rich in polysaccharides, saponins, and flavonoids—exerts immunomodulatory, antioxidant, and metabolic regulatory effects in livestock. While phenotypic improvements in meat quality following *Astragalus membranaceus* supplementation have been reported in small ruminants, the underlying molecular mechanisms remain largely unexplored. In this study, we integrated proteomic and metabolomic analyses to investigate the molecular responses of the *longissimus dorsi* muscle in Ao-Hu crossbred rams to graded dietary supplementation with *Astragalus membranaceus* powder.

### 4.1. Phenotypic Effects of AMP on Meat Quality

Phenotypically, the reduction in cooking loss was significant only in the 6% AMP group relative to the control (*p* < 0.05), whereas no statistically significant differences were observed in meat color, shear force, pH, drip loss, or pressure loss among treatments. This pattern is suggestive of a phenotype-specific response of lamb meat quality to dietary AMP, with the significant effect occurring only at the 6% inclusion level [16]. The improvement in cooking loss may plausibly reflect alterations in protein thermal stability and water-binding capacity during heat-induced denaturation [17], as opposed to changes in free water migration within the myofibrillar network, the latter being more closely associated with drip and pressure losses [18]. For traits such as meat color and pH, however, a potential modulatory effect of AMP cannot be definitively discounted [19]. One possible interpretation is that measurements taken exclusively at 45 min and 24 h postmortem may not have captured the critical window of dynamic metabolic fluctuations [20,21]. Alternatively, the Ao-Hu crossbred genotype employed herein may possess a comparatively high intrinsic antioxidant capacity, which could attenuate the detectable impact of exogenous supplementation [22]. Based on the average daily feed intake data reported in our companion study under identical experimental conditions, the average daily dry matter intakes were 1.33, 1.34, 1.41, and 1.45 kg/d for the C, L, M, and H groups, respectively [3]. Accordingly, the estimated daily AMP intakes were approximately 0, 26.8, 56.4, and 87.0 g/d per lamb for the four groups, respectively. The phenotypic and molecular changes observed exclusively in the H group indicate that a relatively high daily AMP intake was required to elicit detectable effects on meat quality and muscle metabolism.

### 4.2. Metabolic Reprogramming Induced by AMP

Metabolomic profiling was performed to characterize the metabolic alterations in the *longissimus dorsi* muscle following graded AMP supplementation. A total of 24, 33, and 86 differential metabolites were identified in the low-, medium-, and high-dose groups, respectively. KEGG enrichment analysis revealed distinct pathway profiles across the three treatment groups. In the low- and medium-dose groups, differential metabolites were mainly enriched in phenylalanine-tyrosine-tryptophan biosynthesis, lysine biosynthesis, and partial degradation pathways. Notably, the high-dose group exhibited a pronounced pathway shift. In addition to the above amino acid metabolism, significant enrichment was observed in arginine-proline metabolism, pantothenate and CoA biosynthesis, and cAMP/cGMP signalling pathways. This transition is indicative of a more global metabolic reprogramming triggered by AMP bioactives at the higher inclusion level. Coenzyme A (CoA) is a ubiquitous cofactor central to cellular energy metabolism, participating in fatty acid oxidation and the tricarboxylic acid (TCA) cycle [23]. Arginine serves as a precursor for nitric oxide, polyamines, and creatine, and its metabolism participates in collagen synthesis and microcirculation regulation via nitric oxide production [24]. Meanwhile, the cAMP signalling pathway functions as a key hub in energy metabolism, potentially influencing postmortem energy status and protein phosphorylation [25]. The results indicated that the high-dose AMP supplementation significantly activated these pathways, which may be associated with the improved water-holding capacity observed in this treatment group. However, as these pathways were inferred from enrichment analysis without functional validation, this interpretation should be considered hypothesis-generating rather than a demonstrated mechanism. Collectively, these findings suggest that substantial metabolic network changes were observed only at the highest AMP inclusion level (6%), which may be associated with the improved meat quality attributes observed in this treatment group. It should be noted that several pathways enriched in the H group (e.g., Parkinson disease, pertussis, alcoholism) are annotated as human disease pathways in the KEGG database. These annotations do not indicate that ovine muscle manifests these diseases; rather, they reflect conserved molecular pathways—such as oxidative phosphorylation, immune signalling, and neurotransmitter metabolism—that are shared across mammals. Thus, these enrichments should be interpreted as evidence of metabolic network modulation rather than disease-specific events.

### 4.3. Proteomic Responses and the Discrepancy with Metabolomic Findings

In contrast to the metabolome, the proteomic response to AMP supplementation was relatively modest, with 11, 7, and 19 differentially expressed proteins (DEPs) identified in the low-, medium-, and high-dose groups, respectively, and no significant KEGG enrichment reaching statistical significance in the high-dose group. This disparity may reflect the slower turnover of structural proteins relative to small metabolites [26], or it may indicate that AMP bioactives primarily affect the small-molecule metabolic network without substantially altering protein abundances through long-term accumulation, as suggested by recent multi-omics investigations of Astragalus polysaccharides [27,28]. The DEPs identified across the three comparison groups involved structural proteins, metabolic enzymes, and extracellular matrix components, with collagen family members (*COL1A1*, *COL1A2*, and *COL3A1*) showing a consistent downward trend exclusively in the high-dose group. Paradoxically, however, this down-regulation of collagen subunits was not accompanied by a corresponding reduction in shear force values. This seemingly contradictory result could be attributable to two main factors. First, shear force-determined tenderness depends not only on absolute collagen content, but to a greater extent on collagen cross-linking density and thermal solubility [29]. Since neither cross-linking status nor thermal solubility was assessed in the present study, the observed reduction in collagen abundance does not necessarily imply a proportional decrease in mechanical resistance. Thus, although the proposed explanation is biologically plausible, it remains speculative in the absence of direct experimental evidence. The relaxin signalling pathway, which was enriched exclusively in the low-dose group, has been reported to suppress collagen synthesis [30]; however, the absence of this pathway enrichment at higher doses leaves its relevance to the high-dose collagen reduction unclear. Second, tenderness is a multifactorial trait in which myofibrillar protein degradation—particularly of titin and nebulin—plays a dominant role during the early post-mortem period, whereas collagen-related effects on texture become more manifest only with extended aging [31,32]. Since these myofibrillar proteins were not identified as DEPs and the 24 h aging period may have been insufficient for collagen remodelling to translate into detectable shear force changes, the down-regulation of collagen alone would not be expected to alter tenderness within the current experimental timeframe.

### 4.4. Integrated Multi-Omics and Associations with Meat Quality

To further explore the relationship between the observed molecular alterations and AMP supplementation, a Spearman correlation network was constructed to integrate proteomic and metabolomic data with meat quality traits. In the high-dose group cooking loss showed positive correlations with glutathionylspermidine, 5′-deoxyadenosine, prolylphenylalanine, palmitoylcarnitine, and phytosphingosine, and negative correlations with naphthalene epoxide, isobutyl isothiocyanate, FA 16:3;O, and meperidine. Among these, palmitoylcarnitine, as an intermediate of fatty acid β-oxidation [33], may indirectly reflect enhanced fatty acid oxidation within the muscle. This could affect the thermal phase transition properties of membrane lipid bilayers and heat-induced water loss, as thermally induced structural phase transitions have been shown to drive lipid remodeling and oxidation in muscle tissue during cooking [34], and membrane phospholipid deterioration is recognized as a key mechanism underlying water loss in food systems [35]. The thermal response of phospholipid bilayers is critically dependent on fatty acid chain length and saturation [36], and changes in the physical state of muscle cell membranes have been directly linked to water loss during thermal processing [37]. Glutathionylspermidine, a conjugate involved in cellular redox balance, has been associated with antioxidant defense in muscle tissue [38,39]. For the low- and medium-dose groups, although numerous correlations were identified between metabolites and meat quality traits, the absence of corresponding phenotypic changes precludes meaningful biological interpretation; these data are therefore presented without further mechanistic elaboration.

### 4.5. Limitations

Several limitations of this study should be acknowledged. First, this study focused exclusively on the *longissimus dorsi* muscle. Whether the observed AMP-induced molecular changes are generalizable to other muscle types—such as biceps femoris or semimembranosus—remains unknown and warrants further investigation. Second, the study evaluated molecular changes at a single time point at the end of the 70-day feeding period; sampling at multiple time points would provide information on the temporal progression of these responses. Third, as noted throughout this discussion, the correlational nature of the multi-omics integration precludes causal inference, and the observed associations may be influenced by unmeasured confounding variables. Fourth, given that only one conventional meat quality trait (cooking loss) changed significantly among the traits measured, it cannot be concluded that the observed molecular alterations necessarily translated into biologically meaningful improvements in meat quality. Despite these limitations, the present study provides an integrated proteomic and metabolomic view of the ovine muscle response to graded AMP supplementation and identifies distinct metabolic and protein signatures associated with the different inclusion levels that warrant targeted validation in future studies.

## 5. Conclusions

This study provides an integrated proteomic and metabolomic characterization of the *longissimus dorsi* muscle response to graded AMP supplementation in Ao-Hu crossbred rams. Phenotypically, only the 6% AMP inclusion significantly reduced cooking loss, while other traits remained unchanged. Metabolomic analysis identified 24, 33, and 86 differential metabolites in the L, M, and H groups, with the H group showing additional enrichment in arginine-proline metabolism, pantothenate/CoA biosynthesis, and cAMP/cGMP signaling. Proteomic analysis revealed 11, 7, and 19 DEPs, with collagen family members down-regulated exclusively in the H group, though shear force was unaffected. Correlation analysis suggested associations between cooking loss and metabolites linked to fatty acid oxidation and redox balance. These findings provide molecular clues for understanding the effects of AMP on meat quality, with the most pronounced effects observed at the highest inclusion (6%) level. However, given that only one meat quality trait changed significantly and no functional validation was performed, these mechanistic interpretations remain hypothetical and warrant confirmation through targeted metabolite validation, gene expression analyses, and functional assays in future studies.

## Figures and Tables

**Table 1:** Effect of Astragalus powder on meat quality.

Items		C	L	M	H	*p*-Value
Meat color (45 min)	a*	15.56 ± 1.16	14.55 ± 0.40	13.33 ± 4.11	17.55 ± 3.08	0.72
	b*	5.33 ± 0.84	4.56 ± 0.11	4.22 ± 2.07	6.78 ± 1.09	0.513
	L*	7.22 ± 1.75	7.11 ± 0.67	7.78 ± 4.73	11.11 ± 4.78	0.829
Meat color (24 h)	a*	19.00 ± 2.37	17.78 ± 0.55	16.22 ± 0.97	15.78 ± 1.31	0.433
	b*	7.45 ± 1.35	7.89 ± 1.06	6.66 ± 0.88	6.11 ± 0.40	0.606
	L*	20.56 ± 5.68	16.11 ± 5.29	19.33 ± 4.86	14.11 ± 6.64	0.844
Shear force (N)		94.70 ± 6.87	99.93 ± 9.94	92.44 ± 4.45	102.89 ± 5.17	0.709
pH (1 h)		6.49 ± 0.10	6.28 ± 0.06	6.16 ± 0.06	6.26 ± 0.11	0.109
pH (24 h)		5.86 ± 0.10	6.15 ± 0.74	6.00 ± 0.41	5.80 ± 0.33	0.948
Drip loss (%)		68.67 ± 9.06	69.00 ± 7.00	51.82 ± 1.74	59.83 ± 6.23	0.275
Press loss (%)		42.00 ± 4.69	44.96 ± 5.40	27.81 ± 1.40	34.26 ± 4.21	0.074
Cooking loss (%)		17.82 ± 1.69 ^a^	16.01 ± 1.97 ^a^	20.96 ± 1.37 ^a^	10.63 ± 0.88 ^b^	0.009

Data are means ± SEM (*n* = 6). Means in a row with different lowercase letters differ significantly (*p* < 0.05). Absence of letters indicates no significant difference (*p* > 0.05). The abbreviations L, M, H and C represent the low-dose group, medium-dose group, high-dose group, and control group, respectively.

## Data Availability

The data that support the findings of this study are available from the corresponding author upon reasonable request.

## Supplementary Materials

The following supporting information can be downloaded at https://www.mdpi.com/article/10.3390/ani16162479/s1, Table S1: Composition and nutrient levels of experimental diets (air-dry basis, %).

## Abbreviations

The following abbreviations are used in this manuscript:

AMP	*Astragalus membranaceus* powder
PCA	Principal component analysis
PLS-DA	Partial least squares-discriminant analysis
OPLS-DA	Orthogonal partial least squares-discriminant analysis
CoA	Coenzyme A
DEPs	Differentially expressed proteins
