# Supplementary material for: Proteomics- and Metabolomics-Based Investigation of the Effects of Astragalus membranaceus Powder on Meat Quality and Molecular Profiles of Longissimus dorsi Muscle in Ao-Hu Crossbred Rams
Source: Animals (Basel). 2026 Aug 10;16(16):2479. doi: 10.3390/ani16162479 (PMC13508872; doi:10.3390/ani16162479)
Supplement: Supplementary file 1 [file animals-16-02479-s001.zip › animals-4465949-supplementary.pdf]

Table S1. Composition and nutrient levels of experimental diets (air-dry basis, %).

Items		C (0%)	L (2%)	M (4%)	H (6%)
Ingredients					
Roughage	Whole corn silage	54.94	54.94	54.94	54.94
	Corn straw	5.99	5.99	5.99	5.99
	Wheat straw	5.99	5.99	5.99	5.99
	Alfalfa hay	7.49	7.49	7.49	7.49
Concentrate	Wheat bran	3.10	2.10	1.10	0.10
	Triticale	0.25	0.25	0.25	0.25
	Corn	12.98	12.98	12.98	12.98
	Soybean meal	5.49	5.49	5.49	5.49
	Rapeseed cake	3.00	3.00	3.00	3.00
Additive	*Astragalus membranaceus* powder	0.00	2.00	4.00	6.00
	NaCl	0.42	0.42	0.42	0.42
	Limestone	0.15	0.15	0.15	0.15
	Premix [1]	0.20	0.20	0.20	0.20
	Total	100.00	100.00	100.00	100.00
Nutrient levels [2]					
	ME（MJ/kg）	11.15	11.12	11.10	11.07
	CP	17.29	17.17	17.33	17.44
	Ca	0.63	0.64	0.65	0.60
	P	0.40	0.38	0.36	0.34
	NDF	49.96	50.33	50.70	51.08
	ADF	17.32	17.58	17.75	17.94

[1] Premix provided the following for per kg of diets: Cu 250 mg; Fe 25 mg; Zn 1250mg; Mn 750 mg; vitamin A 42,500,000 IU/kg premix; vitamin D_3 17,000,000 IU/kg premix; vitamin E 250 g/kg premix.

[2] ME was calculated according to *Chinese Feeding Standard of Meat-Producing Sheep and Goats* (NY/T 816-2021), while other nutrient levels were measured values.

Proximate composition of experimental diets was determined according to Chinese national standards: crude protein by Kjeldahl method (GB/T 6432-2018), calcium (GB/T 6436-2018), total phosphorus by spectrophotometry (GB/T 6437-2018), neutral detergent fiber (GB/T 20806-2022), and acid detergent fiber (NY/T 1459-2022).
